# Ilizarov bone transport for the treatment of fibular osteomyelitis: a report of five cases

**DOI:** 10.1186/s12891-015-0708-x

**Published:** 2015-09-05

**Authors:** Peng Yin, Lihai Zhang, Lining Zhang, Tongtong Li, Zhirui Li, Jiantao Li, Jianfeng Zhou, Qi Yao, Qun Zhang, Peifu Tang

**Affiliations:** Department of Orthopaedics, Chinese PLA General Hospital, No. 28 Fuxin Road, Beijing, 100853 P.R. China; Medical College, Nankai University, No. 94 Weijin Road, Tianjin, 300071 P.R. China; Department of Orthopaedics, Beijing Shijitan Hospital, Beijing, P.R. China

## Abstract

**Background:**

The objective of this study was to evaluate the effectiveness of the treatment of fibular osteomyelitis by Ilizarov bone transport.

**Methods:**

We retrospectively reviewed 5 patients with fibular osteomyelitis treated by Ilizarov bone transport. Our study included 4 males and 1 female with a mean of age 29.2 years. The average length of the bone defects after radical debridement was 7.6 cm (range 6.5-10 cm).

**Results:**

The mean follow-up was 24.8 months (range 14–34 months). No patient was lost to follow-up. All the patients had bone union and no recurrence of infection was observed. The mean external fixation time was 8.8 months (range 8-10 months), and the mean external fixation index was 1.18 months/cm (range 0.90-1.43 months/cm). There was no sign of knee or ankle instability by clinical examination in all the patients. According to Association for the Study and Application of the Method of Ilizarov (ASAMI) classification, bone results were excellent in 3 patients, good in 2 patients; functional results were excellent in 3 patients, good in 2 patients.

**Conclusions:**

Our study suggested that Ilizarov bone transport may be a good choice for the treatment of fibular osteomyelitis, especially for the patient with distal fibular loss.

## Background

Fibular osteomyelitis is rare in clinical practice, but some intractable problems usually complicate the osteomyelitis including persistent infection, bone necrosis, soft tissue loss and fracture instability [1, 2]. At present, the common treatment for fibular osteomyelitis is partial fibulectomy. Compared with other operative treatment options, partial fibulectomy has some advantages. It can offer effective solution without the uncertainty of bone union following operative fixation or bone grafting. In addition, it does not require the implantation of orthopedic hardware, which can decrease the surgical time, expense and complexity of the treatment [1–5]. However, several clinical and biomechanical studies have reported that partial resection of the fibula can impair knee stability, disturb ankle kinematics significantly and cause weakness of the deep muscles [6–9]. Therefore, in order to get a better treatment result, restoration of fibular integrity maybe necessary.

Ilizarov bone transport can treat osteomyelitis of any length with a less invasive and more versatile compared to other methods. It has been successfully used in the treatment of tibia, femur and upper limbs [10–13]. Nevertheless, to our best knowledge, there is no report about fibular osteomyelitis treated by Ilizarov bone transport. Therefore, we described our successful experience in the treatment of fibular osteomyelitis by Ilizarov bone transport in the following report.

## Methods

Between January 2004 and January 2013, 8 patients with fibular osteomyelitis were treated in our institution. Our eligible criteria were: (1) patients of age of 18 years or more; (2) patients with proximal or distal fibular osteomyelitis treated by Ilizarov bone transport; (3) patients without an associated permanent nerve injury of the ipsilateral lower extremity. At last, 5 patients were included in our study. Our study was retrospective and it didn’t have any harm to our patients. Before performing the surgery, we informed every patient of all possible results that would happen during and after the surgery, and all the patients agreed to receive the surgery and signed the written consent for the surgery. We got the verbal informed consent from the 5 patients through telephone before performing the retrospective study, and our study was approved by the Chinese PLA General Hospital committee for clinical research.

There were 4 males and 1 female with an average of 29.2 years (range 20–39 years). The mechanisms of initial injury involved traffic accident in 3 patients and falling in 2 patients. The site of fibular osteomyelitis involved 4 distal parts and 1 proximal part. The average length of bone defects was 7.6 cm (range 6.5-10 cm), which were measured in the operation. All the fibula infection was caused by iatrogenic operation. Fibular osteomyelitis existed at the time of surgery and the mean number of previous operations was 2.2 (range 1–3 operations). Infecting samples that were obtained from purulent drainage or deep bone at the site of osteomyelitis were cultured and the results were 4 patients with infecting organism of Staph. aureus and 1 patient with Pseudomonas. Further details were listed Table 1.

**Table 1:** Characteristics of 5 patients with fibular osteomyelitis

Case number	Sex	Age (years)	Mechanisms of initial injury	Site of fibular osteomyelitis	Bone defect (cm)	Number of previous operations	Infecting organism
1	Male	20	F	Distal	10	3	Staph. aureus
2	Male	28	TA	Distal	7.5	2	Staph. aureus
3	Female	30	F	Proximal	6.5	3	Staph. aureus
4	Male	35	TA	Distal	7	1	Pseudomonas
5	Male	33	TA	Distal	7	2	Staph. aureus

*F* falling; *TA* traffic accident

### Surgical technique

The patients were positioned supine on a radiolucent table. The operative incisions were performed in accordance with previous surgical incisions when possible. Then the infected scarred soft tissue and necrotic bone were debrided radically. Cortical bone bleeding, described as the so-called paprika sign, was accepted as an indication of vital tissue [14]. Representative tissue cultures were obtained from infected tissue for the sake of finding out the infectious bacterium to choose sensitive antibiotics. The assembled Ilizarov external fixator was fixed to the fibular or tibia as the way that Ilizarov rings were placed on the distal and proximal fragments parallelled to their respective joints and the fixed pins were inserted into the same plane and perpendicular to the mechanical axis of the fibular or tibia under the image intensifier control; A 1–2 cm incision was made in order to expose the pre-selected osteotomy site, and then a subperiosteally transverse osteotomy was performed. The periosteum was sutured and the incisions were closed with drainage tubes. If the infected site had large soft tissue defects, open dressing changing or vacuum sealing drainage (VSD) were made to close the wound. If the bone defect located in the distal part of fibular, bone transport was performed from the proximal to the distal in the fibular; if the bone defect located in the proximal part of fibular, bone transport was performed from the distal to the proximal in the fibular.

### Post-operative protocol

All patients received a course of sensitive antibiotics for 2 to 4 weeks in intravenous way and were encouraged to partial weight-bearing with crutches, isometric muscle and joint range-of-motion exercises on the second day after operation. The latency period before bone transport was 7–10 days and the rate of distraction was 0.25 mm per 6 h. When bone transport completed, the fibular docked ends were compressed by 0.25 mm per day in order to provide full contact until the patient felt pain at the docking site.

Radiographs were reviewed every 2 weeks during the distraction period and monthly during the consolidation period. Ilizarov external fixator was removed when radiographs showed solid docking-site union and the regenerate area had a minimum of three complete cortices. The flexion and extension of knee joint, and the plantarflexion and dorsiflexion of ankle joint were evaluated at the end of follow-up. Bone results and functional results were evaluated according to Association for the Study and Application of the Method of Ilizarov (ASAMI) classification [15–17].

## Results

**Fig. 1:**
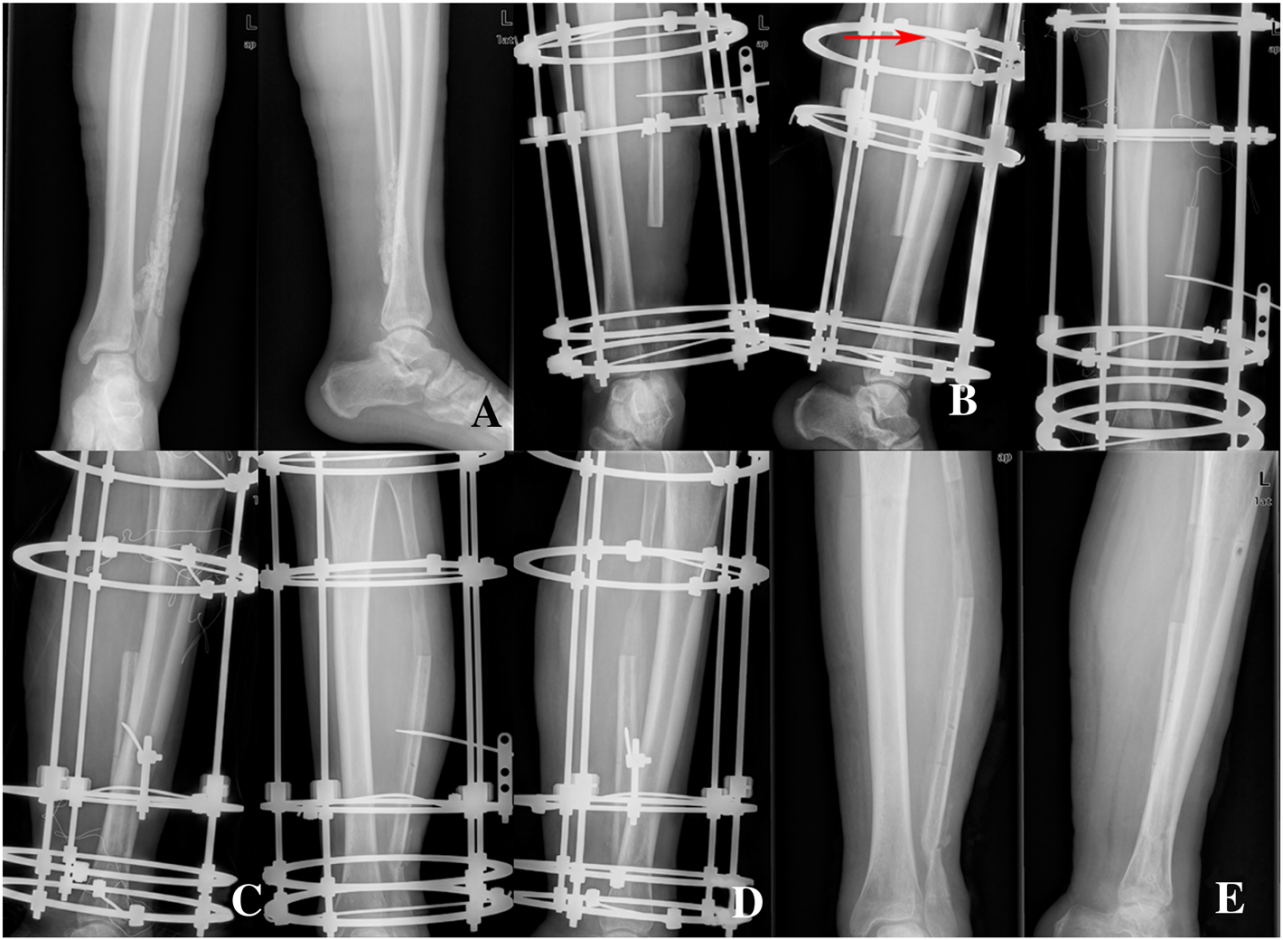
A 20-year-old man with osteomyelitis of the left fibular was treated by Ilizarov bone transport. **a** Radiograph of a 20-year-old man (case 1) who had osteomyelitis of the left fibular. **b** Debridement of the site of infected nonunion with 10cm bone defect in fibular and corticotomy of fibular (red arrow) after application of the fixator. **c** Four months after operation with bone transport, bone ends contacted at the docking site. **d** Nine months after operation, it showed good consolidation of the regenerate, and bone union at the docking site was obtained by bone grafting. **e** The external fixator was removed after nine months of the surgery, and it showed complete bone union

The mean follow-up was 24.8 months (range 14–34 months). No patient was lost to follow-up. All the patients had bone union and no recurrence of infection was observed. The mean external fixation time was 8.8 months (range 8-10 months), and the mean external fixation index was 1.18 months/cm (range 0.90-1.43 months/cm). The mean degrees of knee flexion was 124° (range 120°-130°), and the mean degrees of knee extension was 1° (range 0°-5°). The mean degrees of ankle plantarflexion was 40° (range 35°-45°), and the mean degrees of ankle dorsiflexion was 15° (range 10°-20°). There was no sign of knee or ankle instability by clinical examination in all the patients. All the patients felt that the muscle strength of injured limb was up to the level of contralateral limb muscle. According to Association for the Study and Application of the Method of Ilizarov (ASAMI) classification, bone results were excellent in 3 patients, good in 2 patients; functional results were excellent in 3 patients, good in 2 patients. Further details were listed in Table 2 (Fig. 1).

**Table 2:** Results of treatment in the 5 patients

Case number	External fixation time (months)	External fixation index (months/cm)	Follow-up (months)	Knee flexion/extension (°)	Ankle Plantarflexion/dorsiflexion (°)	Bone result	Functional result
1	9	0.90	24	125-0-0	45-0-15	Good	Excellent
2	8	1.07	30	130-0-0	40-0-10	Excellent	Excellent
3	8	1.23	22	120-0-0	40-0-20	Excellent	Good
4	10	1.43	34	120-0-0	35-0-15	Good	Good
5	9	1.29	14	125-0-5	40-0-15	Excellent	Excellent

### Complications

All the patients had a feeling of pain during the distraction period and required oral analgesics. 4 patients had a pin-track infection (case 1–4), and these patients had only local inflammation, which was treated by pin care and empirical broad spectrum antibiotics for oral administration. 3 patients required bone grafting at the docking site to obtain union (case 1 and case 3–4). There were no neurovascular complications or a compartment syndrome.

## Discussion

This is a retrospective study of Ilizarov bone transport for the treatment of fibular osteomyelitis. The present study showed that the treatment of fibular osteomyelitis by Ilizarov bone transport acquired satisfied results. The kinematics of knee and ankle joint were not disturbed by this treatment, and the motion ranges of knee and ankle joints were nearly normal levels. There was no sign of knee or ankle instability by clinical examination in all the patients. All the patients achieved bone union in the fibular.

It was reported that fibular bore one sixth of the force transmitted from femur [18]. It has an important localization between the knee and ankle joints and contains muscles related with the two joints. The dynamic features of fibular motion and its importance in the clinical have been presented by several studies [6, 19–21]. Leg morbidity after fibular resection has been put forward by some studies [6, 22]. Some clinical and biomechanical studies suggested that the fibular has an important effect on knee stability, ankle kinematics and the strength of deep related muscles [6, 7, 19, 22]. Therefore, our main goals of the treatment of fibular osteomyelitis are to debride infectious tissues radically, regain proper length, and achieve bone union, and obtain better functions of knee and ankle joints. We adopted Ilizarov bone transport to treat fibular osteomyelitis, because it can afford surgeon the ability to treated osteomyelitis and its associated factors simultaneously. In our study, although we got a good treatment result, there were some complications happened. The rate of pin-track infection was 80 % (4/5) and the rate of bone grafting was 60 % (3/5). We recognize that meticulous care, patient’s compliance, and surgeon’s experience are the key factors to decrease the rate of complication.

In our experience, some important aspects should be paid attention in the treatment of fibular osteomyelitis by Ilizarov bone transport. (1) The external fixator should be assembled preoperatively under the image intensifier control in order to decrease the difficulty of reduction. (2) A radical debridement was necessary in the site of fibular osteomyelitis. (3) Particular attention should be paid to protect the integrity of the distal tibiofibular syndesmosis. (4) We should perform bone grafting when the docking site has a delayed union or nonunion.

To the best of our knowledge, this is the first study about fibular osteomyelitis treated by Ilizarov bone transport. The main strength of our study is that all the operations were performed by the same orthopedic surgeon, which can avoid the differences caused by different surgeons’ preference and experience. A number of data on the characteristics of patients, treatment results and complications were reported in our study. However, our study has its limitations. Muscle strength has been assessed subjectively, and there is no objective comparison of the muscle strength. It is retrospective in nature and the number of patients is relatively small, and there is no control group to compare our results with. More prospective randomized controlled trials are needed to overcome the limitations of our study.

## Conclusions

Our study suggested that Ilizarov bone transport may be a good choice for the treatment of fibular osteomyelitis, especially for the patient with distal fibular loss. Restoration of the integrity of distal fibula would be fruitful for ankle stability.

## Abbreviations

ASAMI: Association for the Study and Application of the Method of Ilizarov
